# Evaluation of total choline from in-vivo volume localized proton MR spectroscopy and its response to neoadjuvant chemotherapy in locally advanced breast cancer

**DOI:** 10.1054/bjoc.2000.1711

**Published:** 2001-04

**Authors:** N R Jagannathan, M Kumar, V Seenu, O Coshic, S N Dwivedi, P K Julka, A Srivastava, G K Rath

**Affiliations:** Departments of NMR, Surgery; Biostatistics; Radiotherapy; All India Institute of Medical Sciences, Ansari Nagar, New Delhi, 110029, India

**Keywords:** in-vivo proton magnetic resonance spectroscopy (MRS), locally advanced breast cancer (LABC), neoadjuvant chemotherapy (NACT), preoperative chemotherapy, total choline (TCho)

## Abstract

Results of the proton magnetic resonance spectroscopy carried out on normal, benign breast disease and locally advanced breast cancer patients are presented. The in-vivo MR spectra of malignant breast tissue of patients (*n* = 67) suffering from infiltrating ductal carcinoma are dominated by the water resonance, while the spectra of the unaffected contralateral breast tissue of these patients are mainly dominated by resonance arising from lipids which is similar to the spectra of normal breast tissue obtained from volunteers (controls, *n* = 16). In addition to the water and lipid peaks, in majority of the patients (~80%) the water suppressed spectra showed a resonance at 3.2 ppm due to choline containing compounds (TCho) before treatment. In patients receiving neoadjuvant chemotherapy, absence/reduction in choline was observed in 89% of the patients. TCho was also observed in 2 of 14 benign lesions. The sensitivity and specificity of in-vivo MRS in detecting TCho in malignant tumours was 78% and 86%, respectively. Observation of TCho before treatment and its disappearance (or reduction) after treatment may be a useful indicator of response of locally advanced breast cancer to neoadjuvant chemotherapy. © 2001 Cancer Research Campaign http://www.bjcancer.com


*British Journal of Cancer* (2001) **84**(8), 1016–1022
© 2001 Cancer Research Campaign
doi: 10.1054/ bjoc.2001.1711, available online at http://www.idealibrary.com on IDEAL®

### Evaluation of total choline from in-vivo volume localized proton MR spectroscopy and its response to neoadjuvant chemotherapy in locally advanced breast cancer

**NR Jagannathan[1], M Kumar[1], V Seenu[2], O Coshic[2], SN Dwivedi[3], PK Julka[4], A Srivastava[2] and GK Rath[4]**

Departments of NMR[1], Surgery[2], Biostatistics[3] and Radiotherapy[4], All India Institute of Medical Sciences, Ansari Nagar, New Delhi 110029, India

**Summary** Results of the proton magnetic resonance spectroscopy carried out on normal, benign breast disease and locally advanced breast cancer patients are presented. The in-vivo MR spectra of malignant breast tissue of patients ($n$ = 67) suffering from infiltrating ductal carcinoma are dominated by the water resonance, while the spectra of the unaffected contralateral breast tissue of these patients are mainly dominated by resonance arising from lipids which is similar to the spectra of normal breast tissue obtained from volunteers (controls, $n$ = 16). In addition to the water and lipid peaks, in majority of the patients (~80%) the water suppressed spectra showed a resonance at 3.2 ppm due to choline containing compounds (TCho) before treatment. In patients receiving neoadjuvant chemotherapy, absence/reduction in choline was observed in 89% of the patients. TCho was also observed in 2 of 14 benign lesions. The sensitivity and specificity of in-vivo MRS in detecting TCho in malignant tumours was 78% and 86%, respectively. Observation of TCho before treatment and its disappearance (or reduction) after treatment may be a useful indicator of response of locally advanced breast cancer to neoadjuvant chemotherapy. © 2001 Cancer Research Campaign http://www.bjcancer.com

**Keywords**: in-vivo proton magnetic resonance spectroscopy (MRS); locally advanced breast cancer (LABC); neoadjuvant chemotherapy (NACT); preoperative chemotherapy; total choline (TCho)

Breast cancer is the commonest cancer among women the world over. In India, the reported age adjusted incidence of breast cancer in women is about 26.7/1 00 000 population (NCRP, 1992). Unlike the West, in India, majority of the patients (about 60%) present with locally advanced breast cancer (LABC) or disseminated disease (Goel et al, 1995). In LABC, the treatment policy followed at most centres, is neoadjuvant chemotherapy (NACT) followed by surgery and local or locoregional radiotherapy. The response to NACT is assessed by clinical evaluation and supplemented by radiological measurement of reduction in tumour volume by mammography and/or ultra sonography. None of the currently available indicators of response (clinical and radiological) correlate well with the actual response as assessed on histopathological examination of the tumour.

MR imaging (MRI) is a valuable new tool for diagnostic mammography (Orel et al, 1996; Friedrich, 1998; Harms, 1998; Orel, 1998). Recently, dynamic contrast enhanced MRI techniques have also been developed for differentiation between benign and malignant tumours (Kaiser, 1991; Harms et al, 1993; Haywang-Kobrunner et al, 1997; Piccoli, 1997; Daniel et al, 1998). The above techniques, however, do not provide any metabolic/ biochemical information. On the contrary, magnetic resonance spectroscopy (MRS) permits non-invasive detection of metabolic (biochemical) differences between tumours and normal tissues, and can also be used to monitor response to different treatment regimens. Recently, we have shown that in LABC, the assessment of response to NACT can be made using water-to-fat ratio calculated from volume localized proton MRS (Jagannathan et al, 1998, 1999). In addition, we also reported the presence of choline in a majority of the breast cancer patients (Jagannathan et al, 1998).

In this study, results of evaluation of choline in LABC and its response to NACT using in-vivo proton MRS are presented. The objectives are: (i) to evaluate the potential of proton MRS in the study of breast cancer, and (ii) to investigate further, the recent observation of choline containing compounds in malignant breast tissues and its response to NACT. To the best of our knowledge, this is the first report assessing the response of breast cancer to NACT in a large cohort of patients using in-vivo proton MRS.

### PATIENTS AND METHODS

### Patients

67 women with cytologically confirmed infiltrating ductal carcinoma (IDC) were recruited. Necessary clearance from the Institute ethical committee and written informed consent were obtained prior to examination from patients and controls. Patients were evaluated clinically and tumour size was measured using Vernier calipers. Metastatic workup included liver function tests, chest

*Received 16 November 1999*
*Revised December 2000*
*Accepted 19 January 2001*

*Correspondence to:* NR Jagannathan

A portion of this work was presented at the 7th scientific meeting of the International Society for Magnetic Resonance in Medicine, Philadelphia, Pennsylvania, USA, May 1999.

**Table 1** Summary of patient data

| TNM stage | No. of patients | Menopausal status | | Av. age (yrs) | Tumour size[a] ($cm^2$) | MRS data | | Chemotherapy regimen | | |
|---|---|---|---|---|---|---|---|---|---|---|
| | | Pre | Post | | | Voxel size (ml) | LW (Hz) | CAF | CMF | Pac + Epi |
| $T_2N_2/T_3N_0$ | 7 | 4 | 3 | 46.6 ± 7.6 | 12–22 | 1–8 | 18.1 ± 3.9 | 5 | 1 | 1 |
| $T_3N_1/N_2$ | 7 | 5 | 2 | 44.1 ± 7.8 | 9–52 | 3.4–8 | 10.8 ± 5.9 | 5 | 1 | 1 |
| $T_{4b}N_0$ | 19 | 12 | 7 | 46.1 ± 10.8 | 14–56 | 3.4–8 | 15.6 ± 6.0 | 11 | 5 | 3 |
| $T_{4b}N_1/N_2$ | 34 | 20 | 14 | 42.1 ± 11.8 | 8–144 | 2.2–27 | 9.7 ± 4.0 | 26 | 7 | 1 |

CMF = Cyclophosphamide, Methothrexate & 5-Flurouracil (5FU). All drugs given on 1st and 8th day of a 28 days cycle; CAF = Cyclophosphamide, adriamycin (epirubicin/doxorubicin) and 5-FU. Cyclophosphamide and 5FU given on day 1 and day 8 of a 28 day cycle and doxorubicin on day 1 only; Pac+EPi = Paclitaxal and epirubicin. All drugs given on day 1 of 21 days cycle. [a]Tumour size as determined from clinical evaluation. The data represents the length × breadth (minimum and maximum).

roentgenograms and ultrasound evaluation of the abdomen. Only patients with locally advanced breast cancer were included in this study. The relevant patient data are presented in Table 1. None of the patients had previously been treated with either hormone, chemotherapy or radiotherapy. Treatment protocol used was NACT followed by surgery with local/locoregional radiotherapy. All patients (of Groups II and III) received either 3 or 6 cycles of chemotherapy and the details of the treatment schedule are as given in Table 1. Patients were re-evaluated 2 weeks after the completion of chemotherapy and the response to therapy was assessed clinically including measurement of tumour size. MRS was performed on: normal volunteers (controls, $n = 16$); Group I: 32 patients investigated one week before NACT only; Group II: 21 patients investigated one week after the completion of the 3rd or 6th cycle of NACT only; and Group III: 14 patients who were followed sequentially (one week before therapy and one week after completion of the 3rd or 6th cycle of NACT). One patient was breast feeding at the time of MRS. In 18 patients, proton spectra were also recorded from the unaffected contralateral breast (pretherapy patients, 14 from Group I and 4 from Group III). In addition, 14 cases with benign breast lesion were also studied.

Fine needle aspiration biopsy (FNAB) samples ($n = 3$) from Group I patients were collected in polypropylene vials containing phosphate-buffered saline in $D_2O$ and immediately frozen in liquid nitrogen. The samples were thawed, transferred into 5 mm NMR tubes (~500 µl aliquots) and water suppressed proton spectra was recorded at 400 MHz at 37˚C on the same day. Tumour specimens from 6 patients of Group I were collected and perchloric acid extract (PCA) of the specimen was prepared according to the previously described procedure (Jagannathan and Sendhilvelan, 1993). Spectra of FNAB specimen and PCA extract of biopsy tumour tissues were carried out mainly to confirm the presence of TCho observed in in-vivo MRS.

### MRI/MRS measurements

MRI/MRS was performed at 1.5 Tesla (MAGNETOM, Siemens) using a standard bilateral surface receiver coil provided by the manufacturer. The subjects were positioned prone with each breast fitting into a cup of the surface coil, while the body coil was used as transmitter. Following the scout image, $T_1$-weighted sagittal images were obtained using standard spin-echo sequence and fat suppressed MR images in the transverse and coronal planes. Using these images and depending on the tumour size, voxels of appropriate dimension (Table 1) were chosen and positioned well within the tumour for further MRS study.

In-vivo localized MRS was carried out using the STEAM sequence (Frahm et al, 1987). Magnetic field shimming was carried out both globally and over the voxel region prior to MRS. Line-widths (LW) after voxel shimming corresponded typically to 10–25 Hz for the lipid peak in case of normal/control breast and 5–20 Hz for the water peak in patients with breast tumours. 32 to 64 scans with and without water suppression were collected using an echo time TE = 135 ms and a repetition time TR = 3 s, with the total acquisition time being around 2 to 4 minutes. The free induction decays were zero filled to 4 K data points with a Gaussian broadening of 3 Hz before Fourier transformation. Chemical shifts were reported using water as internal standard at 4.70 ppm. Only the presence or absence of TCho is reported in this study and no objective statistical criteria of the signal-to-noise ratio were used for detection of TCho signal. Investigators performing MRS (NRJ, MK) were not blinded to the pre-treatment clinical diagnosis. However, the presence or absence of total choline resonance was based on strict experimental criteria adopted, namely: (i) the LW of the unsuppressed water peak to be around 5 to 20 Hz, and (ii) the ratio of the water suppression ≥ 20. If these 2 criteria were not met, the data was discarded. The total study time per patient, including imaging and spectroscopy, was between 60 and 75 minutes.

Proton spectra of the FNAB (at 37˚C) and the PCA extracts (at 25˚C) were recorded at 400.13 MHz (Bruker, DRX). Chemical shifts were referenced to an external TMS and $D_2O$ was used as a solvent.

### Data analysis

To compare the proportions between 2 groups of patients, Fisher's Exact Test was used. Pre- and post-therapy status of Group III patients in relation to the presence of choline was compared using McNemar's test. Results were considered significant at 5% level of significance ($P < 0.05$). To assess the sensitivity and specificity of TCho before treatment in relation to histopathology, we grouped pre-therapy patients of Groups I and III and compared with 14 benign cases (fibroadenoma). Since the response to NACT is seen to be effective at the end of 3rd cycle (Jagannathan et al, 1998), the data of patients who had 3 cycles and 6 cycles of therapy were grouped together for the purpose of analysis.

### RESULTS AND DISCUSSION

The proton spectrum (without water suppression – Figure 1A) from an 8 ml voxel (Figure 1B) of normal breast tissue of a

© 2001 Cancer Research Campaign

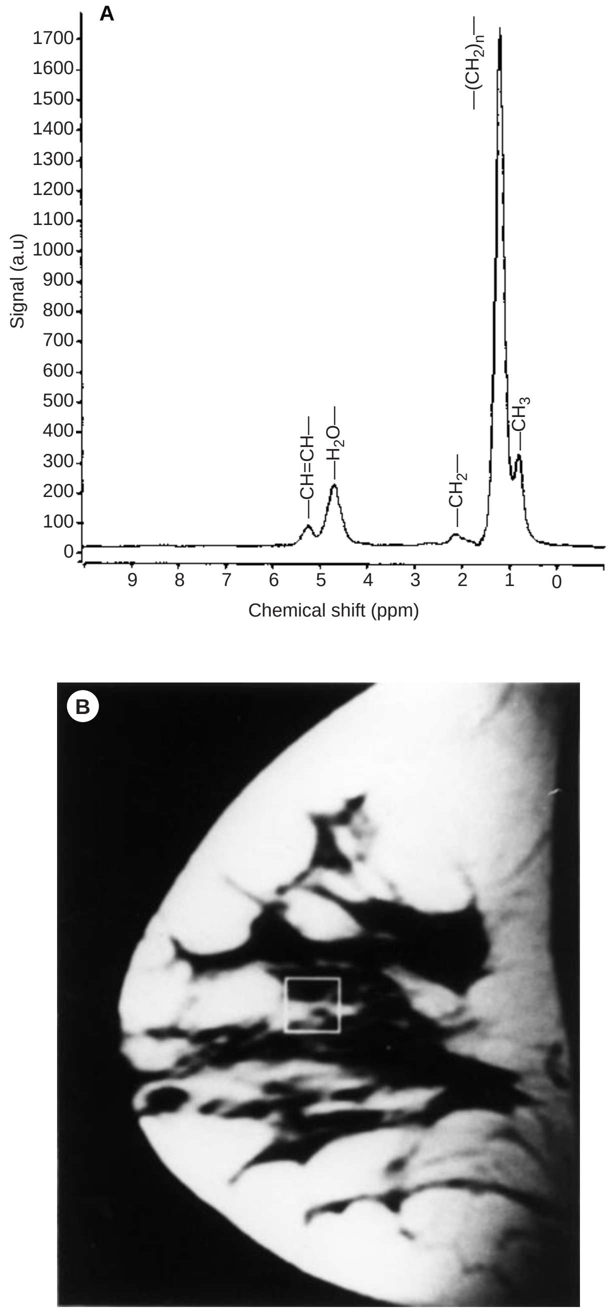

**Figure 1** (**A**) In-vivo localized proton MR spectrum acquired at TE = 135 ms from an 8 ml voxel of 31-year-old normal female volunteer. Resonance assignments are as follows: terminal methyl protons of glycerides at 0.9 ppm, methylene [—$(CH_2)_n$—] protons of lipids at 1.3 ppm, methylene protons $\alpha$ to carboxyl of glyceride chain at 2.2 ppm, diallylic $CH_2$ protons at 2.5 ppm, olefinic hydrogens and CH of glycerol backbone of lipids at 5.2 ppm, water at 4.7 ppm. (**B**) Spin-echo $T_1$-weighted sagittal MR image showing the voxel location

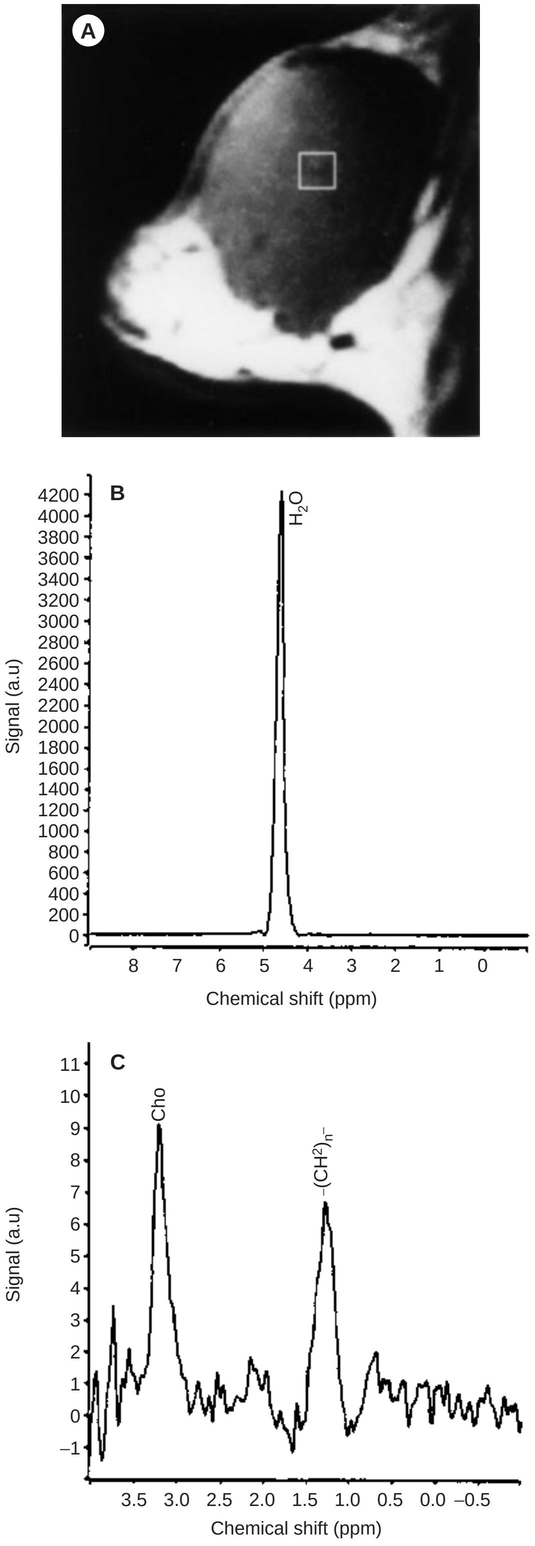

**Figure 2** (**A**) $T_1$-weighted sagittal MR image showing the voxel location. (**B**) Proton MR spectrum at TE = 135 ms from the tumour (8 ml voxel) of a 65-year-old female (#43) suffering from infiltrating duct carcinoma. (**C**) Water suppressed proton spectrum from an 8 ml voxel at TE = 135 ms of the same patient showing choline resonance at 3.2 ppm volunteer shows that resonances from lipid protons dominate. Detailed assignments of other peaks are as given in the figure caption. The spectrum shown in Figure 1 for a control subject is also typical of spectra obtained from the contralateral unaffected breast for all patients. However, it is observed that the spectra depend on the distribution of amount of glandular and fatty breast tissue inside the voxel. With increasing age, the amount of glandular breast tissue decreases and hence, young women were selected as volunteers in the present study to achieve spectra from glandular tissue as well as fatty breast tissue.

*© 2001 Cancer Research Campaign*

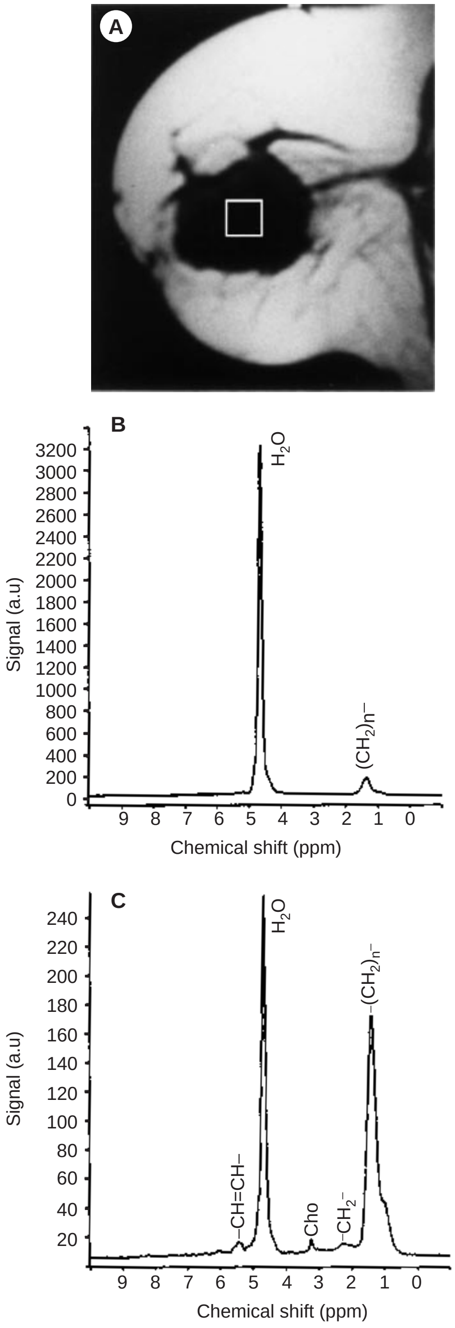

**Figure 3** (**A**) $T_1$-weighted sagittal MR image showing the voxel location. (**B**) Proton MR spectrum at TE = 135 ms from the tumour (8 ml voxel) of a 49-year-old female (#70) suffering from infiltrating ductal carcinoma of the breast. (**C**) Water suppressed spectrum at 135 ms echo time

Figures 2B and 3B show the representative unsuppressed proton spectra from an 8 ml voxel of 2 IDC patients (voxel location in Figures 2A and 3A). The spectra are seen to be different from the normal breast tissue, the water peak dominating with a much lower contribution from lipid protons. This observation is in line with the general hypothesis that tumours have considerably higher water content (Sijens et al, 1988; Gilligam et al, 1997; Jagannathan et al, 1998, 1999; Roebuck et al, 1998). Recently, we documented elevated water-to-fat ratios (W/F) in malignant breast tissues compared to normal breast tissues which showed a statistically significant reduction in patients receiving NACT (Jagannathan et al, 1998, 1999).

Figures 2C and 3C show the water suppressed spectra from an 8 ml voxel of the same patients. In addition to the residual water and fat, a peak at 3.2 ppm due to choline-containing compounds, is clearly seen. In few patients ($n$ = 4, and in one volunteer), other minor resonance in the 8 to 9.5 ppm region were also observed (figure not shown). These were assigned to purine (ATP and GTP) and pyrimidine (uridine and cytidine phosphates) nucleotides. The presence of choline and the assignment of other minor resonances were verified with the help of in-vitro proton spectra of PCA extract of the breast tumor tissues (figure not shown) and FNAB samples. Only the presence or absence of total choline is reported in this paper following strict experimental criteria, as discussed previously. It is our experience that with the use of such strict experimental criteria, the quality of MR spectra obtained markedly improved (with good signal-to-noise ratio), facilitating easy observation of total choline peak. Necessary experimental precautionary measures as outlined earlier, were taken since the presence of total choline may be affected by poor quality local shim, the relative position of the voxel in relation to the surface coil sensitivity and the size of the voxel used.

To evaluate the utility of in-vivo MRS, spectra were recorded for 25 patients (pre-therapy patients of Groups I and III) choosing different regions of the breast which included both tumour and non-tumour region. Figure 4A shows the typical unsuppressed MR spectrum from a voxel which is shifted away (Figure 4B) from tumour. The spectrum looks similar to Figure 1A of normal volunteer, indicating that this region contains normal breast tissue. In addition, no choline was detected in patients ($n$ = 11) where the residual water signal was suppressed. These exercises confirmed that the spectra recorded, reflect the pathological state of the tissue and further validate the observation of choline in malignant breast tissue. 2D/3D chemical shift imaging experiments should further help in discriminating between normal and diseased portions of the breast (Doyle et al, 1999).

Table 2 presents group specific data with desired statistical analysis. Accordingly, TCho was observed in 81% of the Group I patients. For Group II, in 3 out of 21 cases (i.e. 14%), TCho was observed. Table 3 presents the individual data of Group III patients who were monitored sequentially. Total choline was observable in 10 out of 14 cases before treatment. Out of these 10, 7 showed no or significantly reduced TCho, indicating good response to chemotherapy as evidenced by clinical and histopathological evaluation (see Table 3). 3 patients showed no histopathological response to chemotherapy, however, MRS showed significantly reduced TCho in one (#69) and no TCho signal in the other 2 patients (#70 and 81). In another patient (#77) TCho was not observed before treatment, but was detected at the end of 3rd cycle. This anomally could not be rationalized at this point. The post-therapy histopathological investigations of Group III patients correlated well (~80% –11 out of 14 showed concordance) with the presence or absence of choline (Tables 3 and 4). Rapid decrease of phosphomonoesters (one of these is phosphocholine) has been observed in $^{31}$P MRS study of breast carcinoma during effective chemotherapy (Glaholm et al, 1989; Leach et al, 1998).

Further statistical analysis revealed that pre-therapy patients of Groups I and III are comparable in relation to the presence of TCho ($P$ = 0.31). Similarly, the post-therapy status of patients in relation to the absence of TCho are comparable between Groups II and III ($P$ = 0.67). The presence of TCho before initiation of NACT was compared with the histopathological diagnosis (Table 4). Of the 14 benign lesions studied, only 2 showed choline.

© 2001 Cancer Research Campaign 

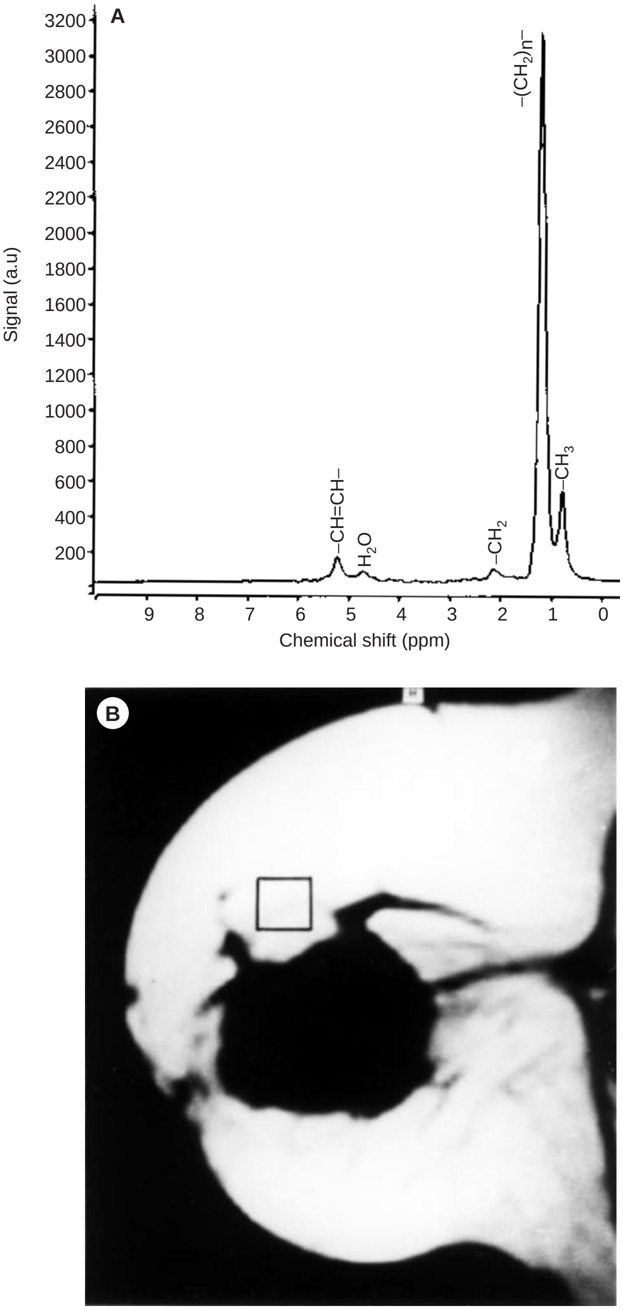

**Figure 4** (**A**) Proton spectrum acquired at an echo time of 135 ms from an 8 ml voxel shifted away from tumour of the same patient shown in Figure 3. (**B**) The corresponding voxel location in the $T_1$-weighted sagittal MR image

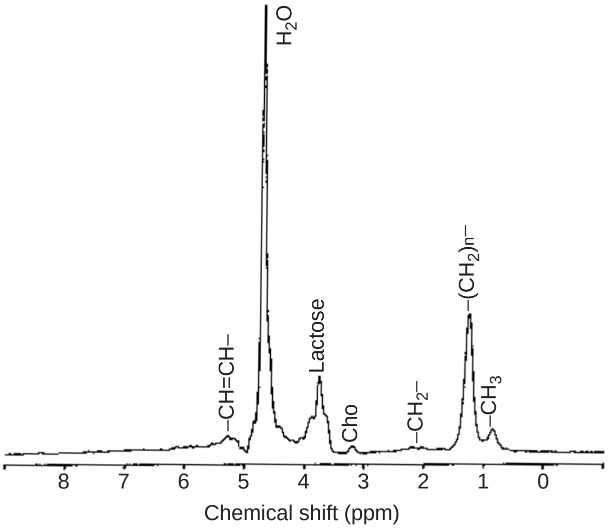

**Figure 5** Proton spectrum from the unaffected contralateral normal breast tissue of a patient who was breast feeding at the time of MRS

The sensitivity of in-vivo MRS in detecting TCho was 78% and the specificity was 86%. In comparison, contrast-enhanced MRI has a high sensitivity (93–99%) but a lower specificity (37–85%) for detecting the breast cancer (Harms et al, 1993; Bone et al, 1997; Heywang-Kobrurner et al, 1997). However, the advantage of in-vivo MRS is that it provides the biochemical/metabolic information which is not available from contrast MRI.

An interesting observation of this study, is the presence of TCho from the contralateral unaffected breast of a patient who was lactating at the time of MRS. Figure 5 shows TCho as well as lactose peak around 3.8 ppm in this patient. Recently, Gribbestad et al (1998) and Kvisted et al (1999) have also observed choline in the normal breast of lactating women as well in 2 out of 11 benign lesions. An increase in phosphomonoester peak in lactating women has also been documented by Twelves et al (1994) through $^{31}$P MRS. Payne et al (1994) have documented that the level of phosphomonoesters changes significantly in normal breast tissues during menstrual cycle. Absolute concentration of choline in breast lesions through in-vivo proton MRS have been determined (Roebuck et al, 1998). Mackinnon et al (1997) reported elevation of choline levels in malignant breast tumours compared to benign cases from in-vivo NMR of FNAB samples and evaluated its sensitivity and specificity in distinguishing benign lesions from invasive cancer. Of the various choline containing compounds that contribute to the peak at 3.2 ppm in in-vivo MRS (choline, glycerophosphocholine and phosphocholine), an increase in

**Table 2** Group specific distribution of patients in relation to the presence of choline along with its percentage and 95% confidence interval (CI)

| | Groups | | | |
|---|---|---|---|---|
| | I | II | III | |
| | Pre | Post | Pre | Post |
| Number of patients with choline | 26 | 3 | 10 | 1 |
| Number of patients with significantly reduced or no choline | 6 | 18 | 4 | 13 |
| % of patients with choline | 81 | 14 | 71 | 7 |
| 95% Confidence | 68–95 | 0–29 | 48–95 | 0–18 |

 © 2001 Cancer Research Campaign

**Table 3** Individual clinical and MRS data of Group III patients who were monitored sequentially

| S. No. | Patient No. | Age (yrs) | Tumour stage | Tumour size (cm) | Presence of choline | | Clinical response | Histopath. response |
|---|---|---|---|---|---|---|---|---|
| | | | | | Pre | Post | | |
| 1 | 43 | 35 | $T_{4b}N_2M_0$ | 9 × 8 | Yes | No | R | R |
| 2 | 44 | 28 | $T_{4b}N_2M_0$ | 8 × 8 | Yes | No | R | R |
| 3 | 47 | 35 | $T_{4b}N_1M_0$ | 11 × 8 | Yes | Yes ↓ | R | R |
| 4 | 50 | 35 | $T_3N_2M_1$ | 8 × 6.5 | No | No | R | R |
| 5 | 60 | 55 | $T_3N_1M_0$ | 6 × 4.5 | Yes | Yes ↓ | R | R |
| 6 | 61 | 30 | $T_{4b}N_2M_0$ | 3.4 × 2.3 | No | No | R | R |
| 7 | 67 | 66 | $T_{4b}N_1M_0$ | 5 × 5 | No | No | R | R |
| 8 | 69 | 26 | $T_{4b}N_2M_1$ | 12 × 12 | Yes | Yes ↓ | NR | NR |
| 9 | 70 | 33 | $T_{4b}N_2M_0$ | 6 × 5 | Yes | No | NR | NR |
| 10 | 77 | 23 | $T_{4b}N_1M_0$ | 8 × 7 | No | Yes | R | R |
| 11 | 79 | 43 | $T_{4c}N_2M_0$ | 18 × 17 | Yes | No | R | R |
| 12 | 81 | 40 | $T_{4b}N_1M_0$ | 9 × 8 | Yes | No | NR | NR |
| 13 | 85 | 32 | $T_{4b}N_2M_0$ | 7 × 6.5 | Yes | No | R | R |
| 14 | 88 | 40 | $T_{4b}N_2M_0$ | 8 × 6 | Yes | No | R | R |

R = corresponds to response to chemotherapy; NR = corresponds to no response to chemotherapy.

**Table 4** Comparison of MRS results with histopathology

| Choline from MRS | IDC* (Groups I and III) and benign cases[a] | | After chemotherapy treatment in Group III patients (*n* = 14) | |
|---|---|---|---|---|
| | IDC (pre-therapy) (*n* = 46) | Benign (*n* = 14) | Responders[a] | Non-responders[a] |
| Present | 36 | 2 | 1 | 1 |
| Absent | 10 | 12 | 10 | 2 |

[a]Confirmed by histopathological evaluation. Note: The sensitivity of in-vivo MR spectroscopy was 78% (36 true positive findings of TCho before therapy, out of 46 total findings), the specificity was 89% [12 true negative findings (benign cases) of 14 total findings].

phosphocholine is highly probable (Katz-Brull et al, 1998; Roebuck et al, 1998).

The phosphocholine (PC) and phosphoethanolamine (PE) are the precursors in the synthesis of phosphatidylcholine (PCho) and phosphatidylethanolamine (PEth), respectively, and are also degradation products of phospholipid breakdown by phospholipase C. Several NMR studies have revealed high concentrations of phosphomonoesters (PC and PE) in human breast tumors (Sijens et al, 1988; Merchant et al, 1988; Glaholm et al, 1989; Degani, 1994; Katz-Brull et al, 1998; Leach et al, 1998). Smith et al (1991) have shown a strong association between the proliferation rate of a rat mammary tumour and the PC and GPC content of the tumour. Gribbestad et al (1993, 1994) reported that PCho showed a large variation between the same type of tumours suggesting breast tumours might have very different choline content. This may be the likely reason for choline to be detected in only 80% of patients studied by us before treatment. Recently, Singer et al (1995) have observed ~18-fold increase in phosphocholine content in 2 primary breast cancer lines (21PT and 21NT), and a 27-fold increase in the metastatic breast cancer cell line (21MT-2) compared with the normal breast epithelial cell line 76N. This increase was accounted for by a decrease in the CTP: cytidylyltransferase activity and/or by increase in choline kinase activity (Merchant et al, 1988). The metastatic breast cancer cell line 21MT-2 also has a significantly higher concentration of PC than do the primary breast cancer cell lines. Recently, Katz-Brull et al (1998) have documented through NMR a biochemical basis for the presence of high phosphocholine in breast carcinoma relative to benign tumours and normal breast tissues.

### CONCLUSIONS

In conclusion, our study demonstrates the utility of volume localized in-vivo proton MR spectroscopy in the study of locally advanced breast cancer. The important finding of this study is the observation of TCho in 78% of the patients prior to therapy. In patients receiving NACT, absence/reduction of TCho was observed in 89% of the patients (31 out of 35 cases of Groups II and III showed no or reduced TCho – see Tables 2 and 3). Our results indicate that the detection of TCho in malignant tumours from in-vivo proton MRS has a good sensitivity (~80%) and specificity (86%). Further studies involving methods to quantitate TCho might be of value for differentiation of breast tumours. Such observations open up the possibility of assessing noninvasively the changes in the concentration of the individual metabolites and their relation with the tumour behaviour, progression, pathophysiology and treatment. The potential clinical use of in-vivo MRS in the management of a patient with breast cancer especially in preoperative diagnosis needs further evaluation. The results presented here, however, have shown that this technique is useful to assess the response of LABC to neuoadjuvant chemotherapy. In

*© 2001 Cancer Research Campaign*

addition, as discussed earlier, at our centre a large number patients present with LABC and accurate assessment of the response to treatment by MRS may help in selecting patients for breast conservation.

### ACKNOWLEDGEMENTS

The authors thank Dr Ian CP Smith and Dr Ingrid S Gribbestad for many helpful discussions, suggestions and critical evaluation of the manuscript. Financial support from the Department of Science and Technology, Government of India (SP/SO/B-27/95) is gratefully acknowledged. We wish to thank Mr M Saxena for typographical assistance.

We wish to dedicate this article in memory of our colleague and co-author of this paper [Dr O Coshic] who met with an untimely death due to AML.

### REFERENCES

Bone B, Pentek Z, Perbeck L and Veres B (1997) Diagnostic accuracy or mammography and contrast enhanced MR imaging in 238 histological verified breast lesions. *Acta Radiol* **38**: 489–496

Daniel BL, Yen YF, Glover GH, Ibeda DM, Birdwell RL, Glover AMS, Black JW, Plevritis SK and Herflens RJ (1998) Breast disease: dynamic spinal MR imaging. *Radiology* **209**: 499–509

Degani H (1994) In: *NMR in Physiology and Biomedicine*. New York: Academic Press, pp 329–351

Den Hollander JA, Luyten PR, Marien AJH, Segebarth CM, Baleriaux DF, De Beer R and Va Ormondt D (1989) Potentials of qualitative image localized human $^{31}$P nuclear magnetic resonance spectroscopy in clinical evaluation of intracranial tumours. *Mag Reson Quat* **5**: 152–168

Doyle VL, Barton SJ and Griffiths JR (1999) $^{31}$P and $^{1}$H MRS of human cancer. *Curr Sci* **76**: 772–776

Frahm J, Merboldt KD and Hanicke W (1987) Localized proton spectroscopy using stimulated echoes. *J Magn Reson* **72**: 502–508

Friedrich M (1998) MRI of the breast: state of the art. *Eur J Radiol* **8**: 707–725

Gilligam P, Flangan FL, Redmond OM, Walsh D, Carney DN and Ennis JT (1997) Proton magnetic resonance spectroscopy in MR mammographic evaluation of breast cancer. *Proc Int Soc Magn Reson Med* **5**: 1378

Glaholm J, Leach MO, Collins DJ, Mansi J, Sharp JC, Madden A, Smith IE and McCready VR (1989) In-vivo $^{31}$P magnetic resonance spectroscopy for monitoring treatment response in breast cancer. *Lancet* **10**: 1326–1327

Goel AK, Seenu V, Shukla NK and Raina VK (1995) Breast cancer presentation at a regional cancer center. *Natl Med J India* **8**: 6–8

Gribbestad IS, Fjosne HE, Haugen OA, Nilsen G, Krane J, Petersen SB and Kvinnsland S (1993) In vitro proton NMR spectroscopy of extracts from human breast tumours and non- involved breast tissue. *Anticancer Res* **13**: 1973–1980

Gribbestad IS, Nilsen G, Fjosne HE, Kvinnsland S and Krane J (1994) NMR spectroscopic characterization of perchloric acid extracts from breast carcinoma and non-involved breast tissue. *NMR Biomed* **7**: 182–196

Gribbestad IS, Singstad TE, Nilsen G, Fjosne HE, Engan T, Haugen OA and Rinck PA (1998) In-vivo $^{1}$H MRS of normal breast and breast tumours using a dedicated double breast coil. *J Magn Reson Img* **8**: 1191–1197

Harms SE, Flemig DP, Hesley KL, Meiches MD, Jensen RA, Evans WP, Savino DA and Wells RV (1993) MR imaging of the breast with rotating delivery of excitatation off resonance: clinical experience with pathologic correlation. *Radiology* **187**: 493–501

Heywang-Kobrunner SH, Viehuray P, Heining A and Kuchler C (1997) Contrast-enhanced MRI of the breast; accuracy, value, controvarsies, solutions. *Eur J Radiol* **24**: 94–108

Jagannathan NR and Sendhilvelan S (1993) Therapeutic response of tumors by in-vitro proton nuclear magnetic resonance spectroscopy. *Appl Magn Reson* **5**: 357–367

Jagannathan NR, Meenakshi Singh, Govindaraju V, Raghunathan P, Coshic O, Julka PK and Rath GK (1998) Volume localized in-vivo proton MR spectroscopy of breast carcinoma: Variation of W/F ratio in patients receiving chemotherapy. *NMR Biomed* **11**: 414–421

Jagannathan NR, Mahesh Kumar, Raghunathan P, Coshic O, Julka PK and Rath GK (1999) Assessment of the therapeutic response of human breast carcinoma using in-vivo volume localized proton magnetic resonance spectroscopy. *Curr Sci* **76**: 777–782

Kaiser WA (1993) MR mammography, *Radiologie* **33**: 292–299 and *Mdica Mundi* **36**: 168–182, 1991

Kasiomos JN, Merchant TE, Gierke LW and Glonek T (1990) $^{31}$P magnetic resonance spectroscopy of human colon cancer. *Cancer Res* **50**: 527–532

Katz-Brull R, Margalit R, Bendel P and Degani H (1998) Choline meatbolism in breast cancer; $^{2}$H, $^{13}$C and $^{31}$P NMR studies of cells and tumors. *MAGMA* **6**: 44–52

Kvistad KA, Bakken IJ, Gribbestad IS, Ehrnholm B, Lundgren S, Fjosne HE and Haraldseth O (1999) Characterization of neoplastic and normal human breast tissue with in-vivo $^{1}$H MR spectroscopy. *J Magn Reson Img* **10**: 159–164

Leach MO, Verrill M, Glaholm J, Smith TA, Collins DJ, Payne GS, Sharp JC, Ronen SM, McCready VR, Powles TJ and Smith IE (1998) Measurements of human breast cancer using magnetic resonance spectroscopy: a review of clinical measurements and a report of localized $^{31}$P measurements of response to treatment. *NMR Biomed* **11**: 314–40

Mackinnon WB, Barry PA, Malycha PL, Gillett DJ, Russell P, Lean CL, Doran ST, Barraclough BH, Bilous M and Mountford CE (1997) Fine-needle biopsy specimens of benign breast lesions distinguished from invasive cancer ex-vivo with proton MR spectroscopy. *Radiology* **204**: 661–666

Merchant TE, Gierke LW, Meneses P and Glonek T (1988) $^{31}$P Magnetic resonance spectroscopic profiles of neoplastic human breast tissues. *Cancer Res* **48**: 5112–5118

NCRP (National Cancer Registry Programme) Biennial Report 1988–1989 An Epidemiological Study, Indian Council of Medical Research: New Delhi, 1992

Orel SG (1998) High-resolution MR imaging for the detection, diagnosis, and staging of breast cancer. *Radiographics* **18**: 903–912

Orel SG, Hochman MG, Schnall MD, Reynolds C and Sullivan DC (1996) High-resolution MR imaging of the breast: clinical context. *Radiographics* **16**: 1385–1401

Payne GS, Dowsett M and Leach MO (1994) Harmone dependent metabolic changes in the normal breast monitored noninvasively by $^{31}$P MR spectroscopy. *Breast* **3**: 20–23

Piccoli CW (1997) Contrast-enhanced breast MRI: factors affecting sensitivity and specificity. *Eur J Radiol* 7 (suppl.5): 281–288

Roebuck JR, Cecil KM, Schnall MD and Lenkinski RE (1998) Human breast lesions: Characterization with proton MR spectroscopy. Radiology **209**: 269–275

Sijens PE, Wijrdeman HK, Moerland MA, Bakker CJG, Vermeulen JWA and Luyten PR (1988) Human breast cancer in vivo: H-1 and P-31 MR spectroscopy at 1.5 T. *Radiology* **169**: 615–620

Singer S, Souza K and Thilly WG (1995) Pyruvate utilization, phosphocholine and adenosine triphosphate (ATP) are markers of human breast tumor progression: A $^{31}$P and $^{13}$C Nuclear Magnetic Resonance (NMR) spectroscopy study. *Cancer Res* **55**: 5140–5145

Smith TAD, Eccles S, Ovmerod MG, Tombs AJ, Titley JC and Leach MO (1991) The phosphocholine and glycerophosphocholine content of an esterogen sensitive rat mammary tumor correlates strongly with growth rate. *Br J Cancer* **64**: 821–826

Twelves CJ, Porter DA, Lowry M, Dobbs NA, Grover PE, Smith MA, Rubens RD and Richards MAC (1994) Phosphorus-31 metabolism of post-menopousal breast cancer studied in vivo by magnetic resonance spectroscopy. *Br J cancer* **69**: 1151–1556

*© 2001 Cancer Research Campaign*

## References

[PDF_00654] Boné B., Péntek Z., Perbeck L., Veress B. (1997). Diagnostic accuracy of mammography and contrast-enhanced MR imaging in 238 histologically verified breast lesions.. Acta Radiol.

[PDF_00657] Daniel B. L., Yen Y. F., Glover G. H., Ikeda D. M., Birdwell R. L., Sawyer-Glover A. M., Black J. W., Plevritis S. K., Jeffrey S. S., Herfkens R. J. (1998). Breast disease: dynamic spiral MR imaging.. Radiology.

[PDF_00673] Friedrich M. (1998). MRI of the breast: state of the art.. Eur Radiol.

[PDF_00677] Glaholm J., Leach M. O., Collins D. J., Mansi J., Sharp J. C., Madden A., Smith I. E., McCready V. R. (1989). In-vivo 31P magnetic resonance spectroscopy for monitoring treatment response in breast cancer.. Lancet.

[PDF_00681] Goel A. K., Seenu V., Shukla N. K., Raina V. (1995). Breast cancer presentation at a regional cancer centre.. Natl Med J India.

[PDF_00683] Gribbestad I. S., Fjösne H. E., Haugen O. A., Nilsen G., Krane J., Petersen S. B., Kvinnsland S. (1993). In vitro proton NMR spectroscopy of extracts from human breast tumours and non-involved breast tissue.. Anticancer Res.

[PDF_00690] Gribbestad I. S., Singstad T. E., Nilsen G., Fjøsne H. E., Engan T., Haugen O. A., Rinck P. A. (1998). In vivo 1H MRS of normal breast and breast tumors using a dedicated double breast coil.. J Magn Reson Imaging.

[PDF_00694] Harms S. E., Flamig D. P., Hesley K. L., Meiches M. D., Jensen R. A., Evans W. P., Savino D. A., Wells R. V. (1993). MR imaging of the breast with rotating delivery of excitation off resonance: clinical experience with pathologic correlation.. Radiology.

[PDF_00698] Heywang-Köbrunner S. H., Viehweg P., Heinig A., Küchler C. (1997). Contrast-enhanced MRI of the breast: accuracy, value, controversies, solutions.. Eur J Radiol.

[PDF_00705] Jagannathan N. R., Singh M., Govindaraju V., Raghunathan P., Coshic O., Julka P. K., Rath G. K. (1998). Volume localized in vivo proton MR spectroscopy of breast carcinoma: variation of water-fat ratio in patients receiving chemotherapy.. NMR Biomed.

[PDF_00712] Kaiser W. A. (1993). MR-Mammographie.. Radiologe.

[PDF_00714] Kasimos J. N., Merchant T. E., Gierke L. W., Glonek T. (1990). 31P magnetic resonance spectroscopy of human colon cancer.. Cancer Res.

[PDF_00717] Katz-Brull R., Margalit R., Bendel P., Degani H. (1998). Choline metabolism in breast cancer; 2H-, 13C- and 31P-NMR studies of cells and tumors.. MAGMA.

[PDF_00723] Kvistad K. A., Bakken I. J., Gribbestad I. S., Ehrnholm B., Lundgren S., Fjøsne H. E., Haraldseth O. (1999). Characterization of neoplastic and normal human breast tissues with in vivo (1)H MR spectroscopy.. J Magn Reson Imaging.

[PDF_00727] Leach M. O., Verrill M., Glaholm J., Smith T. A., Collins D. J., Payne G. S., Sharp J. C., Ronen S. M., McCready V. R., Powles T. J. (1998). Measurements of human breast cancer using magnetic resonance spectroscopy: a review of clinical measurements and a report of localized 31P measurements of response to treatment.. NMR Biomed.

[PDF_00733] Mackinnon W. B., Barry P. A., Malycha P. L., Gillett D. J., Russell P., Lean C. L., Doran S. T., Barraclough B. H., Bilous M., Mountford C. E. (1997). Fine-needle biopsy specimens of benign breast lesions distinguished from invasive cancer ex vivo with proton MR spectroscopy.. Radiology.

[PDF_00737] Merchant T. E., Gierke L. W., Meneses P., Glonek T. (1988). 31P magnetic resonance spectroscopic profiles of neoplastic human breast tissues.. Cancer Res.

[PDF_00743] Orel S. G. (1998). High-resolution MR imaging for the detection, diagnosis, and staging of breast cancer.. Radiographics.

[PDF_00745] Orel S. G., Hochman M. G., Schnall M. D., Reynolds C., Sullivan D. C. (1996). High-resolution MR imaging of the breast: clinical context.. Radiographics.

[PDF_00752] Piccoli C. W. (1997). Contrast-enhanced breast MRI: factors affecting sensitivity and specificity.. Eur Radiol.

[PDF_00754] Roebuck J. R., Cecil K. M., Schnall M. D., Lenkinski R. E. (1998). Human breast lesions: characterization with proton MR spectroscopy.. Radiology.

[PDF_00757] Sijens P. E., Wijrdeman H. K., Moerland M. A., Bakker C. J., Vermeulen J. W., Luyten P. R. (1988). Human breast cancer in vivo: H-1 and P-31 MR spectroscopy at 1.5 T.. Radiology.

[PDF_00760] Singer S., Souza K., Thilly W. G. (1995). Pyruvate utilization, phosphocholine and adenosine triphosphate (ATP) are markers of human breast tumor progression: a 31P- and 13C-nuclear magnetic resonance (NMR) spectroscopy study.. Cancer Res.

[PDF_00766] Smith T. A., Eccles S., Ormerod M. G., Tombs A. J., Titley J. C., Leach M. O. (1991). The phosphocholine and glycerophosphocholine content of an oestrogen-sensitive rat mammary tumour correlates strongly with growth rate.. Br J Cancer.

[PDF_00770] Twelves C. J., Porter D. A., Lowry M., Dobbs N. A., Graves P. E., Smith M. A., Rubens R. D., Richards M. A. (1994). Phosphorus-31 metabolism of post-menopausal breast cancer studied in vivo by magnetic resonance spectroscopy.. Br J Cancer.

[PDF_00662] den Hollander J. A., Luyten P. R., Mariën A. J., Segebarth C. M., Balériaux D. F., de Beer R., van Ormondt D. (1989). Potentials of quantitative image-localized human 31P nuclear magnetic resonance spectroscopy in the clinical evaluation of intracranial tumors.. Magn Reson Q.

